# Prostaglandin dose and time to fetal expulsion after intrauterine fetal death at 22 to 28 gestational weeks in Sweden: A retrospective cohort study

**DOI:** 10.1002/ijgo.70312

**Published:** 2025-06-27

**Authors:** Fanny Berger, Eva Wiberg Itzel, Irene Sterpu, Margit Endler

**Affiliations:** ^1^ Department of Obstetrics and Gynecology South General Hospital Stockholm Sweden; ^2^ Department of Clinical Science and Education Karolinska Institutet, South General Hospital Stockholm Sweden; ^3^ Division of Obstetrics and Gynecology, Department of Clinical Sciences Intervention and Technology (CLINTEC), Karolinska Institutet Stockholm Sweden; ^4^ Department of Women's and Children's Health Karolinska Institutet Stockholm Sweden

**Keywords:** blood loss, duration of labor, induction of labor, intrauterine fetal death, prostaglandins

## Abstract

**Objective:**

Prostaglandin regimens for induction of intrauterine fetal death (IUFD) <28 gestational weeks (GW) are unstandardized. We aimed to describe prostaglandin regimens for induction after IUFD and assess association between dose and time to fetal expulsion.

**Methods:**

We performed a retrospective cohort study among all singleton IUFD 22–28 GW in the Stockholm region, Sweden, 2008–2015. The cumulative prostaglandin dose within 6 h was categorized as a low‐, medium‐, or high (equivalent to ≤200 μg, >200 μg but <800 μg, or ≥800 μg of misoprostol) based on variations in current clinical guidelines. Our primary outcome was time to fetal expulsion >12 h assessed using multinomial logistic regression adjusting for parity, age, gestational age, Bishop score and previous cesarean section.

**Results:**

Among 136 IUFD cases, 22 prostaglandin induction regimens were used. A total of 51 (77.3%) women in the low‐dose group had an induction >12 h compared to 11 (30.6%) in the medium‐, and 13 (42.4%) in the high, adjusted risk ratio (ARR) 1.91 (95% confidence interval [CI]: 1.43–2.23) between the high‐ and low‐dose group. Median time to fetal expulsion was 21 h 11 min in the low‐ (*P* < 0.001), 10 h 21 min in the medium‐ (*P* = 0.90) and 10 h 12 min in the high‐dose group.

**Conclusion:**

IUFD cases <28 GW induced with doses equivalent to misoprostol ≤200 μg within 6 h on average had significantly longer time to fetal expulsion and were almost twice as likely to have an induction >12 h compared to higher doses. Women with IUFD at this gestation would likely benefit from higher prostaglandin doses than those recommended in clinical guidelines if safety is ascertained.

## 1 INTRODUCTION

Intrauterine fetal death (IUFD) occurs in 3–4 per 1000 births in Sweden and is a cause of enormous distress.^1^, ^2^ The recommended mode of delivery for IUFD is vaginal in the absence of contraindications such as placenta previa or fetopelvic disproportion; however, there is no consensus regarding the optimal prostaglandin dose regimen.^3^, ^4^

The combination of the progesterone antagonist mifepristone and a prostaglandin such as misoprostol or dinoprostone for induction of labor is effective for both mid‐ and late trimester IUFD and have similar safety profiles according to a recent meta‐analysis.^5^, ^6^, ^7^ In the Stockholm region, there is no region‐specific protocol for induction of labor of IUFD and different traditions regarding dose and route of administration coexist.^8^, ^9^, ^10^, ^11^, ^12^ According to a meta‐analysis of 14 randomized controlled trials on induction methods for IUFD in the second and third trimester, this reflects the global scenario, with a wide array of treatment doses and routes of administration.^13^

There is limited research comparing the effectiveness of prostaglandin dose and time intervals for induction of IUFD below 28 gestational weeks (GW). According to the American College of Obstetricians and Gynecologists (ACOG), given limited evidence, the typical dose of misoprostol for induction <28 GW is 400–600 μg vaginally every 3 to 6 h.^4^ A review of misoprostol use for late IUFD presented in the guidelines from the Royal College of Obstetricians and Gynecologists (RCOG), recommends that the dose should be adjusted according to gestational age with 200 μg given 4‐hourly before 28 GW, and 25–50 μg given vaginally 4‐hourly at or above 28 GW.^14^ Empirical evidence suggests that regimens at gestations above 28 GW are more standardized and are managed similarly to inductions of live fetuses whereas the gestational range from 22 to 28 weeks would benefit from further research and standardization.^4^

In Sweden, IUFD is defined as pregnancies that end spontaneously at or above 22 GW but the gestational cutoff for IUFD varies globally. Different definitions, as well as heterogenous dosing and time regimens, have complicated the comparison of effectiveness between induction methods for IUFD. Previous studies and guidelines on IUFD and nonviable pregnancies have shown that further research is needed regarding the best mode of induction to provide adequate guidelines at different gestational age.^15^, ^16^

This study aimed to describe the spectrum of prostaglandin regimens for induction of labor after IUFD at gestations between 22 and 28 GW weeks in the Stockholm region, and assess the association between prostaglandin dose, duration of labor and blood loss.

## 2 MATERIALS AND METHODS

We performed a retrospective cohort study of all inductions of labor after IUFD performed using prostaglandins below 28 GW at six obstetric hospitals in the Stockholm region between 2008 and 2015. Patient data containing demographic‐, maternal background‐, pregnancy‐ and birth‐related information were recorded prospectively and extracted retrospectively for the study in pseudonymized form from electronic hospital records.

We included all singleton pregnancies with IUFD induced between gestational age 22 weeks 0 days and 27 weeks 6 days. We excluded cases with multiple pregnancies, spontaneous onset labor, delivery by cesarean section, induction initiated with amniotomy or cervical dilation, intrapartum fetal death, and those with missing data for method of induction.

Our main exposure was prostaglandin dose. Dose was assessed based on the total prostaglandin dose administered within the first 6 h of the induction either vaginally, orally or sublingually, and categorized as low, medium or high. The high‐dose category attained or exceeded the misoprostol dose cited by the ACOG consensus,^4^ the low adhered to the current RCOG guidelines,^17^ and the medium category were all doses in between those two regimens. Low dose was therefore ≤200 μg misoprostol, medium doses were >200 μg but <800 μg misoprostol, and high doses were ≥800 μg misoprostol. Dinoprostone 1 mg, 2 mg and slow‐release 10 mg (0.3 mg/h) were assessed as equivalent to 25, 50, and 50 μg misoprostol, respectively in line with previous equivalence research and thus categorized as a low‐dose regimens.^5^ We chose dose within the first 6 h as our exposure because using total prostaglandin dose would mean mixing women with two different types of prostaglandin exposure: those with high starting doses (and hypothetically shorter induction times) and those with cumulatively increasing doses over time, potentially reflecting a lack of effect of the initial low starting doses.

In Sweden doses of misoprostol are given 3‐hourly, dinoprostone 1 mg or 2 mg 6‐hourly, and dinoprostone 10 mg (0.3 mg/h slow release) 12‐hourly. All forms of dinoprostone are administered vaginally, misoprostol for induction can be administered orally, sublingually or vaginally. There was no stipulated maximum daily dose for induction of IUFD, but doses would not exceed the maximum daily dose for second trimester abortion which is 2400 mg. Mifepristone 200–600 mg was during the study period routinely given 24–48 h prior to admission for intake of prostaglandin and women received 8.3 μg of oxytocin intramuscularly after delivery to prevent excessive blood loss. If no fetal expulsion occurred within the first day, mifepristone was sometimes repeated after which misoprostol treatment resumed the next day.

Our main outcome was time to fetal expulsion >12 h. Time to fetal expulsion was defined as the duration between the administration of the first dose of prostaglandin and the expulsion of the fetus. Secondary outcomes were median time to fetal expulsion, median blood loss (mL), and postpartum hemorrhage (PPH) defined as bleeding ≥500 mL.

### 2.1 Statistical analysis

We describe categorical data groupwise as numbers and incidence rates. Chi‐square was used to test for differences between groups. Continuous data that did not follow normal distribution are presented as median and interquartile range (IQR) and were compared using analysis of variance (ANOVA). A *P* value less than 0.05 was considered statistically significant. The statistical software SPSS 17.0 was used for data analysis.

We described our main exposure variable, the cumulative dose of prostaglandins given for induction in the first 6 h. We compared medians across dose‐categories using ANOVA. We assessed the association between prostaglandin dose category and our main outcome time to fetal expulsion above 12 h using multinomial logistic regression. The following confounders were adjusted for: maternal age, gestational age, previous cesarean section, Bishop score and parity. The model was predefined to account for variables that might influence both induction dose and labor duration, including those not statistically different between exposure groups, to mitigate any residual confounding. Adjusted odds ratios (AOR) were converted to adjusted risk ratios (ARR) using Zhang's formula to better suit the cohort design and high expected incidence of our main outcome.^18^

Our sample represents all inductions in our setting between 2008 and 2015. Due to the expected heterogeneity in the exposure variable and unknown variance of duration of induction we were not able to assess the power of our primary analysis a priori. Post hoc we determined that if we had expected the risk increase in time to fetal expulsion >12 h in the low‐dose group compared to the high, given the ratio of low to high dose participants, to achieve 90% power and 95% confidence in our findings we would require a total of 105 participants. In a subanalysis, we therefore pooled the high‐ and medium dose groups, between which there was no difference in time to fetal expulsion and compared outcomes in this group to the low‐dose group using binomial logistic regression with OR converted to RR.

The study was approved by the Swedish Ethical Review Authority (registration no.: 2021‐00480).

## 3 RESULTS

**FIGURE 1:**
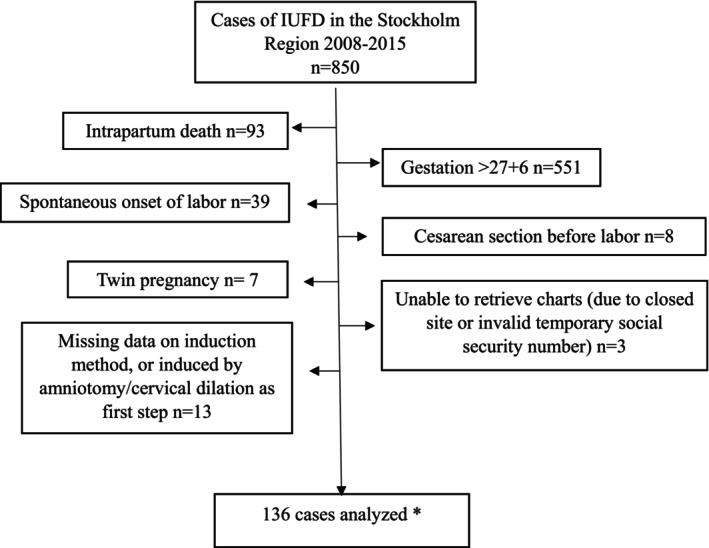
Flow chart of inclusion to the study. *Exclusions are shown in their order of hierarchy, meaning that there may have been cases of multiple pregnancies, that were excluded in a prior category.

Between 2008 and 2015, 850 cases of IUFD occurred in the study setting. The subsequent exclusions resulting in our final dataset are presented in Figure 1 among which were 551 cases ≥28 GW, 93 cases of intrapartum death, 39 cases of spontaneous onset of labor, eight cases with cesarean section before labor, and seven twin pregnancies. Our final study sample consisted of 136 singleton cases of IUFD induced with prostaglandins.

Median maternal age in the low‐, medium‐ and high prostaglandin dose groups was 32, 31, and 31 years, respectively (*P* = 0.255). A total of 10 (12.9%), three (7.7%) and two (7.7%) women in each respective group had a previous cesarean section (*P* = 0.647). Close to half of women in each group were primiparous (*P* = 0.078). Median Bishop score was 1, 1, and 0 (*P* = 0.373). All inductions occurred at a similar median gestational age and fetal weight. All women had vaginal birth after induction except for one who was delivered by vacuum extraction. Background and birth‐related characteristics by induction dose are shown in Table 1.

**TABLE 1:** Background maternal‐, and delivery‐related factors among 136 women induced for intrauterine fetal death between 22 and 28 gestational weeks.

	Prostaglandin dose category for induction			*P* value
	Low^a^ (*n* = 66)	Medium^a^ (*n* = 36)	High^a^ (*n* = 34)	
Maternal age (years), median IQR	32 (29–36)	31 (27–34)	31 (27–35)	0.255
BMI (kg/m^2^), median IQR	25.1 (21.5–28.1)	24.2 (22.8–27.4)	24.1 (21.4–27.5)	0.789
Primiparity, *n* (%)	25 (37.9)	16 (44.4)	21 (61.8)	0.078
Chronic illness,^b^ *n* (%)	9 (13.6)	5 (13.9)	4 (11.8)	0.575
Prior cesarean section, *n* (%)	8 (12.9)	3 (7.7)	2 (7.7)	0.647
Bishop score, median IQR	1 (0–3)	1 (0–1)	0 (0–2)	0.373
Gestational age, median IQR	24 weeks 5 days (23 weeks 4 days–26 weeks 0 days)	24 weeks 6 days (23 weeks 3 days–27 weeks 1 day)	24 weeks 0 days (23 weeks 3 days–25 weeks 6 days)	0.451
Fetal weight (g), median IQR	465 (345–560)	438 (303–693)	530 (355–735)	0.571
Vaginal birth, *n* (%)	65 (98.5%)	36 (100%)	34 (100%)	0.586
Dose of misoprostol in 6 h (μg), median IQR	100 (50–100)	400 (400–600)	1200 (800–1200)	**<0**.**001**

*Note*: BMI, calculated as weight in kilograms divided by the square of height in meters. Missing data: 2 for maternal age, 15 for BMI, 9 for parity, 11 for chronic illness, 62 for Bishop score, 4 for fetal weight. Statistically significant values presented in bold.

Abbreviations: BMI, body mass index; g, grams; h, hours; IQR, interquartile range; *n*, number; μg, micrograms.

^a^ Low‐, medium‐, and high doses equal or equivalent to ≤200 μg misoprostol, >200 μg and <800 μg, and ≥800 μg, respectively within 6 h of the start of the induction.

^b^ Diabetes mellitus, cardiovascular disease, hypertension, lung disease/asthma, chronic kidney disease or endocrine diseases.

Our 136 cases were induced using 22 different two‐dose prostaglandin regimens. Median equivalent misoprostol dose in 6 h was 100 μg (IQR: 50–100), 400 μg (IQR: 400–600), and 1200 μg (IQR: 800–1200) in the low‐, medium‐, and high dose respectively (*P* < 0.001). A total of 48 women were induced with dinoprostone only and 88 with misoprostol only, no women received both misoprostol and dinoprostone in the first 6 h of the induction. A total of 66 were categorized as low‐, 36 as medium‐, and 34 as high‐dose regimens. All dose regimen categories found in the study are presented by dose category in Table 2. Total dose regimens including route of administration of prostaglandins are available in Supplement A. If route of administration, and subsequent prostaglandin doses and interventions after 6 h were considered we found no two identical individual induction regimens.

**TABLE 2:** Modes of induction using prostaglandin regimens among 136 cases of IUFD at 22 to 28 gestational weeks between 2008 and 2015 in Sweden.

Modes of induction		
Low dose^a^	Medium dose^a^	High dose^a^
Misoprostol 25 μg + 25 μg	Misoprostol^b^ 25 μg + 200 μg	Misoprostol 200 μg + 800 μg
Misoprostol 50 μg + 25 μg	Misoprostol 50 μg + 200 μg	Misoprostol 400 μg + 400 μg
Dinoprostone^b^ 1 mg + 1 mg	Misoprostol 50 μg + 400 μg	Misoprostol 600 μg + 400 μg
Dinoprostone 2 mg + 1 mg	Misoprostol 200 μg + 100 μg	Misoprostol 800 μg + 400 μg
Dinoprostone 1 mg + 2 mg	Misoprostol 200 μg + 200 μg	Misoprostol 800 μg + 600 μg
Dinoprostone 2 mg + 2 mg	Misoprostol 100 μg + 400 μg	
Dinoprostone 10 mg slow release	Misoprostol 200 μg + 400 μg	
	Misoprostol 200 μg + 200 μg	
	Misoprostol 200 μg + 400 μg	
	Misoprostol 400 μg + 200 μg	

Abbreviations: IUFD, intrauterine fetal death; μg, micrograms.

^a^ Low‐, medium‐, and high doses equal or equivalent to ≤200 μg misoprostol, >200 μg and <800 μg, and ≥800 μg, respectively within 6 h of the start of the induction.

^b^ A total of 48 women were induced using dinoprostone only in the first 6 h, 88 women were induced using misoprostol only, no women received both misoprostol and dinoprostone in the first 6 h.

A total of 51 women in the low‐dose group (77.3%) had an induction ≥12 h compared to 11 (30.6%) in the medium‐, and 13 (42.4%) in the high‐dose group which corresponded to an ARR of 1.91 (95% confidence interval [CI]: 1.43–2.23) between the low dose and the high dose and an ARR of 0.57 (95% CI: 0.20–1.19) between the medium‐ and high dose group (Table 3).

**TABLE 3:** Time to fetal expulsion and blood loss according to dose of misoprostol among 136 women induced for intrauterine fetal death between 22 and 28 gestational weeks in Sweden.

	Prostaglandin dose category		
	Low^a^	Medium	High
	*n* = 66	*n* = 36	*n* = 34
Time to fetal expulsion (h/min), median IQR	21 h 11 min (13 h 30 min–33 h 41 min)	10 h 21 min (7 h 44 min–13 h 53 min)	10 h 12 min (5 h 10 min–15 h 37 min)
Groupwise comparison of medians, *P* value	**<0.001**	0.841	ref
Induction >12 h^b^, *n* (%)	51 (77.3)	11 (30.6)	14 (42.4)
ARR^c^ 95% CI	**1.91 (1.43–2.23)**	0.57 (0.20–1.19)	ref
Blood loss^b^ (mL), median IQR	200 (150–300)	250 (150–400)	200 (150–300)
Groupwise comparison of medians, *P* value	0.677	0.246	ref
Blood loss ≥500 mL, *n* (%)	7 (12.3%)	6 (20.0%)	4 (13.3%)
ARR^c^ 95% CI	1.59 (0.41–4.16)	0.79 (0.20–2.49)	ref

*Note*: Statistically significant values presented in bold.

Abbreviations: AOR, adjusted odds ratio; CI, confidence interval; h, hours; IQ, interquartile range; min, minutes; *n*, number.

^a^ Low‐, medium‐, and high doses equal or equivalent to ≤200 μg misoprostol, >200 μg and <800 μg, and ≥800 μg, respectively within 6 h of the start of the induction.

^b^ Missing data time to fetal expulsion = 1, blood loss = 19.

^c^ Adjusted for maternal age, parity, gestational age, previous cesarean section, and Bishop score.

Median time to fetal expulsion was 21 h 11 min (IQR: 13 h 30 min–33 h 41 min) in the low‐, 10 h 21 min (IQR: 7 h 44 min–13 h 53 min) in the medium‐, and 10 h 12 min (IQR: 5 h 10 min–15 h 37 min) in the high‐dose groups, respectively. Median time to fetal expulsion was significantly longer in the low‐ compared to the high‐dose group (*P* < 0.001) but not in the medium‐ compared to the high‐dose group (*P* = 0.841).

Median blood loss was 200 mL in the low‐ (IQR: 150–300, *P* = 0.677), 250 mL in the medium‐ (IQR: 150–400, *P* = 0.246), and 200 mL (IQR: 150–300) in the high‐dose group. The rate of PPH was 12.3% (*n* = 7), 20% (*n* = 6) and 13.3% (*n* = 4) and corresponded to an ARR of 1.59 (95% CI: 0.41–4.16) between the low dose and the high dose and an ARR of 0.79 (95% CI: 0.20–2.49) between the medium‐ and high dose group.

In our binary analysis where the medium‐ and high dose groups were merged for increased power (Table 4), time to fetal expulsion in the low‐dose remained double that of the medium/high: 21 h 11 min versus 10 h 17 min, (*P* < 0.001). A total of 51 women (77.3%) in the low dose and 25 (36.2%) in the medium high dose had a time to fetal expulsion above 12 h corresponding to an ARR of 2.43 (95% CI: 1.86–2.66).

**TABLE 4:** Time to fetal expulsion and blood loss according to dose of misoprostol among 136 women induced for intrauterine fetal death between 22 and 28 gestational weeks: A subanalysis with two dose categories.

	Dose of prostaglandin for induction		
	Low^a^	Medium_high^a^	*P* value or adjusted risk ratio^c^
	*n* = 66	*n* = 70	95% CI
Time to fetal expulsion (h/min), median IQR^b^	21 h 11 min (13 h 30 min–33 h 41 min)	10 h 17 min (7 h 17 min–15 h 27 min)	**<0.001**
Induction >12 h, *n* (%)	51 (77.3)	25 (36.2)	**2.43 (1.86–2.66)**
Blood loss (mL), median IQR^b^	200 (150–300)	215 (150–350)	0.763
Blood loss ≥500 mL, *n* (%)	7 (12.3)	10 (16.7)	0.88 (0.30–2.20)

*Note*: Statistically significant values presented in bold.

Abbreviations: AOR, adjusted odds ratio; CI, confidence interval; h, hours; IQR, interquartile range: min, minutes; *n*, number.

^a^ Low‐, and medium_high doses equal or equivalent to ≤200 μg misoprostol, and >200 μg within the 6 h of the start of the induction, respectively.

^b^ Missing data time to fetal expulsion = 1, blood loss = 19.

^c^ Adjusted for maternal age, parity, gestational age, previous cesarean section, and Bishop score.

## 4 DISCUSSION

Our results indicate that a broad spectrum of prostaglandin dose regimens is used to induce patients with IUFD at gestational ages below 28 GW in our setting. Time to fetal expulsion in the group receiving the low‐dose regimen of misoprostol or equivalent was significantly higher than that of the high or medium‐dose groups, as was their risk of having an induction last more than 12 h. Blood loss did not differ by prostaglandin induction dose.

We recorded 22 different induction starting doses for IUFD. If route of administration, prostaglandin doses beyond the first two, and further interventions were accounted for, there were no two individual regimens that were the same. Doses tended to increase as the induction progressed, but dose increases, and administration routes varied seemingly at random. This variation indicates that there is room for a more standardized protocol for this gestational period. The only meta‐analysis of prostaglandin induction for IUFD dates back to 2009 but current guidelines suggest that little had changed in the direction of standardization and that the conclusion that further research is needed that takes into account the different GW is still relevant.^13^

Median duration of induction and the risk of time to fetal expulsion above 12 h among women receiving a low prostaglandin induction dose was twice that of women receiving a medium or high dose. Women in the medium‐dose category received on average 400 μg of misoprostol in the first 6 h whereas the high dose group received 1200 μg, yet time to fetal expulsion between these groups were similar. This suggests prolonged induction is linked to very low doses. The reason induction doses are titrated according to gestational age is because the myometrium grows increasingly sensitive to prostaglandins as the pregnancy progresses.^19^ The dose used for induction of labor in full‐term pregnancies (25 μg) is for example approximately 3% of the initial misoprostol dose used in first trimester abortions (800 μg).^20^, ^21^ At full‐term gestation, a misoprostol dose equivalent to the abortion dose would risk hyperstimulation and at worst uterine rupture, particularly for women with a previous uterine scar.^22^

In clinical guidelines based on studies of induction of IUFD and lethal fetal anomalies at GW 24–28, the highest studied dose, 400 μg misoprostol within 6 h, appeared to be safe but did not reduce time to fetal expulsion.^16^ A systematic review on IUFD induction, including gestations from 14 GW, found no significant difference between 400 μg and 600 μg for fetal expulsion within 24 h, and recommending using the lowest effective regimen. The authors tentatively concluded that doses lower than 400 μg seemed to prolong the time to fetal expulsion.^13^ According to ACOG the recommended dose of misoprostol in second trimester abortions (13–26 weeks) is 400–800 μg as a starting dose,^23^ higher than the typical doses in IUFD at corresponding gestational age.^4^ Our study supports that induction of IUFD requires a treatment aligned with that given when terminating live pregnancies at corresponding gestational age.

Our study showed no significant difference in blood loss between groups, either in median blood loss or rates of PPH. Prolonged labor is, however, associated with PPH which further supports shortening time to fetal expulsions.^24^ In a study on IUFD induction, with prostaglandin doses stratified by gestational age (400 μg from 14 to 26 weeks, 100 μg from 27 to 36 weeks, and 50 μg from 37 to 42 weeks 4‐hourly) there were 21% of cases with hemorrhage ≥500 mL and 7% with hemorrhage ≥1000 mL.^25^ In a pilot study on induction of abortion and IUFD with misoprostol, PPH >1000 mL occurred in 12% of cases.^7^ Our study showed lower rates in all groups, incidence rates which are also lower than the Swedish national rates of PPH and severe PPH among all gestations.^26^

Our study included a limited number of participants from one region and data collection ended in 2015. The variation in current clinical guidelines however indicates that induction methods remain unstandardized and that low‐dose prostaglandin regimens are still used. We had a significant amount of missing data for Bishop score which may have impacted both prostaglandin dose and time to fetal expulsion. Women being induced below 28 GW are, however, likely to have a low Bishop score compared to later in pregnancy when the cervix matures before onset of labor, and we saw no intergroup differences for this factor. We could not take the mifepristone dose or time‐interval into consideration. Studies have shown that pretreatment with mifepristone shortens time to fetal expulsion in IUFD 21–25 weeks^6^ and >24 weeks.^27^ Two systematic reviews of 24 versus 48 h mifepristone to misoprostol interval in abortions in the second trimester concluded that time to fetal expulsion was unaffected by dose interval but a later randomized controlled trial suggested a 48 h interval may shorten induction time.^28^, ^29^, ^30^ We also did not account for route of administration. Pharmacodynamic studies show that although the rise in serum concentration of misoprostol after sublingual, oral, and vaginal administration in the first hour varies, therapeutic interval over 6 h is similar. Further we do not report on side‐effects related to prostaglandin dose or the subjective experience of induction for IUFD in relation to prostaglandin dose and induction time. These parameters must be weighed into any recommendation for induction regimens. In a clinical trial on abortion in the second trimester using our definition of high‐dose misoprostol, chills/shivering, nausea and diarrhea were all been reported to be above 20%.^30^

Our study suggests that women with IUFD between 22 and 28 GW would benefit from higher prostaglandin starting doses to shorten time to fetal expulsion. A shorter time to fetal expulsion may decrease the stress of the experience, and shorten hospital stay with its associated risks. The lowest equally efficient dose should, however, be aimed for to minimize the risk of hyperstimulation and dose‐dependent side‐effects. Further research should determine the effectiveness and subjective experience of a medium compared to high dose regimen for this patient group. There seems, however, no clinical justification for using lower doses of prostaglandin for IUFD than what is used for abortions at the same gestational age.

## 5 CONCLUSIONS

There is a broad spectrum of prostaglandin regimens in use for induction of labor of IUFD at 22 to 28 GW in Sweden. Doses at or below 200 μg in the first 6 h may be associated with a significantly longer time to fetal expulsion and higher likelihood of time to fetal expulsion above 12 h, something which future guidelines may want to consider should further research substantiate these findings.

## AUTHOR CONTRIBUTIONS

Irene Sterpu had the original idea for the study, Eva Wiberg Itzel provided the dataset, Margit Endler, Irene Sterpu, and Fanny Berger developed the analysis plan. Fanny Berger and Margit Endler performed the data analysis and Fanny Berger wrote the first draft of the manuscript. All authors have had access to the data and have approved the final version of the manuscript.

## FUNDING INFORMATION

The study received no external funding.

## CONFLICT OF INTEREST STATEMENT

The authors have no conflicts of interest to report.

## Supporting information


Data S1.

## Data Availability

The data that support the findings of this study are available from the corresponding author upon reasonable request.
