# Supplementary material for: Prostaglandin dose and time to fetal expulsion after intrauterine fetal death at 22 to 28 gestational weeks in Sweden: A retrospective cohort study
Source: Int J Gynaecol Obstet. 2025 Jun 27;172(1):444–50. doi: 10.1002/ijgo.70312 (PMC12724047; doi:10.1002/ijgo.70312)
Supplement: Supplementary file 1 — Data S1. [file IJGO-172-444-s001.docx]

**Supplement A**

**Total doses and route of administration among 136 women induced for intrauterine fetal death between 22 and 28 gestational weeks**

| **Total induction dose and route of administration** | | |
| --- | --- | --- |
| **Miso 2 pox2** | 69 | **Dinopros 2mg x1** |
| **Miso 1 vag** | 70 | **Dinopros 2mgx2** |
| **Miso 4 vag** | 71 | **Miso 1/2 vag+1 vag+1 1/2 vag** |
| **Miso 2 vag** | 72 | **Dinopros 2mg x2 + Oxytocin infusion + Amniotomy** |
| **Miso 4 vag+2 Miso po** | 73 | **Miso 4 vag+Miso 2 po+ Miso 2 vagx3+Amniotomy** |
| **Dinopros 1mg** | 74 | **Dinopros 2mg x4** |
| **Dinopros 2mgx2+Oxytocin infusion** | 75 | **Dinopros 2mgx2+1 Miso vag+Intracervical balloon catheter** |
| **Miso 1 vag x3** | 76 | **Miso 1/8 po x5+ Miso 2 po x3** |
| **Miso 2 pox1+ Intracervical balloon catheter** | 77 | **Dinopros 2 mgx2+Intracervical balloon catheter +2 Miso vag** |
| **Miso 1 po x3** | 78 | **Dinopros 1mgx2+Dinopros 2mg+Intracervical balloon catheter +Oxytocin infusion** |
| **Dinopros 2mg** | 79 | **Dinopros 1mg intracervical + Dinopros 2mg x3 vag + Mife 1 + Miso 3 vag** |
| **Miso 1/8 po x 7+Intracervical balloon catheter** | 80 | **Dinopros 2mg x2 + Dinopros 1mg intracervikalt x2 + Miso 4 vag + OxytocinOxytocin infusion** |
| **Dinopros 1mgx2+Oxytocin infusion** | 81 | **Dinopros 10mg slow release + Dinopros 1mg + Dinopros 2mg + Intracervical balloon catheter + Amniotomy** |
| **Miso 1 vag x 5+ Mife+2 vag x2** | 82 | **Dinopros 2mg + Amniotomy + OxytocinOxytocin infusion** |
| **Miso 1/8 pox 8+Dinopros 2mgx1+Miso 1/2 vag+Oxytocin infusion** | 83 | **Miso 4 vag+2 po x2+Amniotomy** |
| **Miso 1 vagx1+1 po x3+ 2 vag x4** | 84 | **Miso 4 vag** |
| **Miso 2 vagx3+4 Miso vag+2 Miso vagx3+ Oxytocin infusion** | 85 | **Miso 1/4 vag** |
| **Miso 1/4 vag+1/8po x8+1 po x4+Mife+1 Miso** | 86 | **Miso 4 vag+Amniotomy+Oxytocin infusion** |
| **Miso 1/8x8+Dinopros 2mgx2+Intracervical balloon catheter** | 87 | **Miso po 1 + Miso 4 vag + Mife + Amniotomy** |
| **Miso 2 vag x2 +Oxytocin infusion** | 88 | **Miso vag 1 + Miso 2 vag + Miso 3 vag** |
| **Miso 1 vag + Miso 2 vag** | 89 | **Dinopros 10mg slow release** |
| **Dinopros 2mg x2 + Amniotomy** | 90 | **Miso 1/8 po x2, Miso 1 vag x3** |
| **Dinopros 2mg + 1mg Dinopros + Amniotomy + Oxytocin infusion** | 91 | **Miso 1 vag+2 Miso vag+ 3 Miso vag+Oxytocin infusion** |
| **Dinopros 2mgx3** | 92 | **Miso 1/8 po x6** |
| **Dinopros 2mg x2 +Dinopros oklar dos + Dinopros 2mg x2** | 93 | **Miso 1 vag** |
| **Miso 1 vag+1 po** | 94 | **Dinopros 2mg x2 + Intracervical balloon catheter + Oxytocin infusion** |
| **Dinopros oklar dos + 2mg Dinopros + 1mg Dinopros intracervical** | 95 | **Miso 1 vag + Miso 1/2 vag + Miso unclear dose and route + Miso vag unclear dos + Oxytocin infusion** |
| **Miso 4 vag** | 96 | **Miso 1 vag+1 po x 3** |
| **Miso 4 vag + Miso 2 po** | 97 | **Dinopros 2mgx2+Dinopros 1mg** |
| **Miso 1 vag + 2 Miso vag + 1 Miso vag + Amniotomy** | 98 | **Miso 1 vagx2+ 2 Miso vag+3 Miso vag+Amniotomy** |
| **Miso 1/2 vag+ 2 vag+Amniotomy** | 99 | **Miso 4 vag** |
| **Miso 1 vag + Miso 1 vag x3** | 100 | **Miso 1 vag+Amniotomy** |
| **Miso 1 vag x1+ 1 po x1** | 101 | **Miso 1 po** |
| **Miso vag 2x4** | 102 | **Miso 1/4 vag+ 1 Miso vag** |
| **Dinopros 2mg + Intracervical balloon catheter** | 103 | **Miso 4 vag+2 po** |
| **Miso vag 4 + 2 Miso po x2 + 3 Miso vag+ Amniotomy + Oxytocin infusion** | 104 | **Miso 4 route unclear + Miso 2 per os x3** |
| **Dinopros 2 mg x1** | 105 | **Miso 1 vag+ 2 vag x2** |
| **Miso 4 vag +2 po** | 106 | **Propess + Intracervical balloon catheter + Amniotomy + Oxytocin infusion** |
| **Dinopros 2mgx2+Dinopros 1mg+Intracervical balloon catheter +Amniotomy** | 107 | **Dinopros 2mg x4** |
| **Miso 2 po** | 108 | **Dinopros 1mgx2+Dinopros 2mg+Intracervical balloon catheter +Amniotomy+Oxytocin infusion** |
| **Miso 1 po** | 109 | **Dinopros 1mg +Dinopros 1mg+miso dose unclear** |
| **Miso 4 vag + 2 Miso po** | 110 | **Miso 4 vag + Intracervical balloon catheter** |
| **Miso 3 vag + Intracervical balloon catheter + Miso 3 vag + Amniotomy + Oxytocin infusion** | 111 | **Miso 1 vag x1+ 1 po x1+ Dinopros 2 mg x1** |
| **Miso 4 + 2 Miso po + 2 Miso po + Amniotomy** | 112 | **Dinopros 2mgx3+Miso 2 vag+Amniotomy** |
| **Dinopros 1mg + Oxytocin infusion** | 113 | **Miso 4 vag+2 pox2+Intracervical balloon catheter +Amniotomy+Oxytocin infusion** |
| **Miso 1 vag + Miso 2 vag + Miso vag 3** | 114 | **Miso 1 vag+2 vag+3 vagx2+Oxytocin infusion** |
| **Miso 4 vag + Miso 2 po x5 + Amniotomy** | 115 | **Miso 1/2 vag+1 Miso vag x2** |
| **Miso 4 vag+2 pox2** | 116 | **Dinopros 2mg** |
| **Dinopros 2mg+ Dinopros 1mg** | 117 | **Dinopros 2mg x2** |
| **Dinopros 1mg+Dinopros 2mg x2** | 118 | **Dinopros 1mg intracervikalt + Amniotomy + Oxytocin infusion** |
| **Dinopros 2mg x3** | 119 | **Miso 2n vag+1 po x2** |
| **Miso 2 po x3 + Mife 1 po + Miso 2 vag + Oxytocin infusion** | 120 | **Miso 2 vag** |
| **Dinopros 2mg + Dinopros 2mg + Amniotomy + Oxytocin infusion** | 121 | **Miso 1 vag + Miso 1,5 vag + Miso 1 vag** |
| **Dinopros 2mg x3 + Dinopros 1mg intracervical + Amniotomy** | 122 | **Miso 4 vag+ Miso 2 po x2+ Intracervical balloon catheter** |
| **Miso 4 vag + Miso 2 vag + Miso 3 vag + Miso 2 po x5 + 1 Miso po** | 123 | **Miso 1/8 pox5** |
| **Miso 1/8 pox4+2 Miso po x3+Amniotomy+Oxytocin infusion** | 124 | **Dinopros 1mgx2+Intracervical balloon catheter +Dinopros 1mg+Dinopros 2mgx2+ Miso 2x1** |
| **Dinopros 2mgx2+Intracervical balloon catheter +Amniotomy+Oxytocin infusion** | 125 | **Dinopros 2mgx2+Dinopros 1mg+Intracervical balloon catheter** |
| **Dinopros 1mg x2+Intracervical balloon catheter+Amniotomy+Oxytocin infusion+4 vag Miso** | 126 | **Miso 4 vag+2 pot x3+Amniotomy+Oxytocin infusion+2 po+1 po** |
| **Dinopros 2mg x4 + 1 Miso vag + Intracervical balloon catheter + Amniotomy** | 127 | **Miso 2 vag** |
| **Dinopros 2mgx3+Intracervical balloon catheter +Amniotomy+Oxytocin infusion** | 128 | **Miso 4 vag** |
| **Miso vag 1 + Miso vag 1** | 129 | **Dinopros 2mg x2** |
| **Miso 4 vag** | 130 | **Miso 1 vag + Miso 1 unclear route + Miso vag 2 + Amniotomy + oxytocin iv 8,3ug** |
| **Miso 1 vag** | 131 | **Dinopros 2mg x4+ Amniotomy** |
| **Miso 3 vag + Miso 2 po** | 132 | **Dinopros 2mgx2+Dinopros 1mg+Intracervical balloon catheter +Amniotomy+Oxytocin infusion** |
| **Miso 1/4 vag+Miso 1 po** | 133 | **Miso 1 vagx1 + 2 vag x2** |
| **Miso vag 1+2+3** | 134 | **Miso 4 vag + Miso 3 vag and 5 ml NaCl** |
| **Miso 2 vag+1 po x2** | 135 | **Dinopros 2mg + Intracervical balloon catheter + Amniotomy** |
| **Miso 2 unclear route+ 2 Miso 2 vag** | 136 | **Miso 1 vagx2 +2 Miso vag** |

1 Miso = 200ug misoprostol

Abbreviated medications: Miso= misorprostol, Mife= mifepristone, Dinopros= dinoprostone

Abbreviated routes of administration: po=per oral (orally), vag= vaginally
